# 23andMe: a new two-sided data-banking market model

**DOI:** 10.1186/s12910-016-0101-9

**Published:** 2016-03-31

**Authors:** Henri-Corto Stoeklé, Marie-France Mamzer-Bruneel, Guillaume Vogt, Christian Hervé

**Affiliations:** Medical Ethics and Legal Medicine Laboratory EA4569, Paris Descartes University, Centre Universitaire des Saints-Pères, Paris, France; Laboratory of Human Genetics of Infectious Diseases, Necker Branch, Institut National de la Santé et de la Recherche Médicale INSERM-U1163, Paris, France; Paris Descartes University, Imagine Institute, Paris, France; Assistance Publique-Hôpitaux de Paris AP-HP, Necker-Enfants Malades Hospital, Paris, France; CAncer Research For PErsonalized Medicine (CARPEM), Paris Descartes, APHP (HEGP, Cochin, Necker) INSERM, Paris, France

**Keywords:** Biobanking, Data banking, Direct-to-consumer (DTC) genetic testing, Two-sided markets, Research, Service, Ethical issues

## Abstract

**Background:**

Since 2006, the genetic testing company 23andMe has collected biological samples, self-reported information, and consent documents for biobanking and research from more than 1,000,000 individuals (90 % participating in research), through a direct-to-consumer (DTC) online genetic-testing service providing a genetic ancestry report and a genetic health report. However, on November 22, 2013, the Food and Drug Administration (FDA) halted the sale of genetic health testing, on the grounds that 23andMe was not acting in accordance with federal law, by selling tests of undemonstrated reliability as predictive tests for medical risk factors. Consumers could still obtain the genetic ancestry report, but they no longer had access to the genetic health report in the United States (US). However, this did not prevent the company from continuing its health research, with previously obtained and future samples, provided that consent had been obtained from the consumers concerned, or with health reports for individuals from other countries. Furthermore, 23andMe was granted FDA authorization on February 19, 2015, first to provide reports about Bloom syndrome carrier status, and, more recently, to provide consumers with “carrier status” information for 35 genes known (with high levels of confidence) to cause disease.

**Discussion:**

In this Debate, we highlight the likelihood that the primary objective of the company was probably two-fold: promoting itself within the market for predictive testing for human genetic diseases and ancestry at a low cost to consumers, and establishing a high-value database/biobank for research (one of the largest biobanks of human deoxyribonucleic acid (DNA) and personal information).

**Summary:**

By dint of this marketing approach, a two-sided market has been established between the consumer and the research laboratories, involving the establishment of a database/DNA biobank for scientific and financial gain. We describe here the profound ethical issues raised by this setup.

## Background

**Fig. 1:**
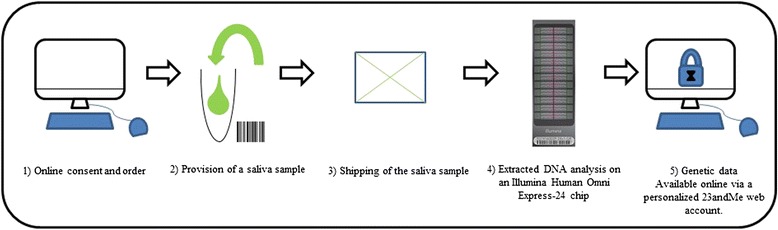
DTC genetic testing service of 23andMe, according to its website

23andMe is a company based in Mountain View, California. This specialist company operates in the biotechnology sector and was founded in 2006 by Linda Avey and Anne Wojcicki. Google has been one of the principal investors in the four rounds of investment in this company (Table 1) [1]. 23andMe offers a direct-to-consumer (DTC) genetic testing service based on the use of single-nucleotide polymorphisms (SNPs) to determine ancestry and to identify genetic markers associated with specific diseases and conditions and a few specific causal variants that the company claimed could provide information about their clients’ health and how to improve it [2]. The approach used can be summarized in five steps, as follows (Fig. 1): 1) online consent and ordering of the kit; 2) delivery of the kit to the client's home for the collection of a saliva sample; 3) shipping of the saliva sample to 23andMe; 4) DNA extraction and analysis on an Illumina Human Omni Express-24 chip (2 million SNPs covering the whole genome); 5) provision of the genetic results online via a personalized 23andMe web account six to eight weeks after reception of the sample by the company. However, DTC health genetic testing was being carried out in the absence of a medical prescription or information from a whole-genome analysis (WGA) based on genome-wide association (GWA) studies. The use of such an approach raised questions about the reliability of the 23andMe test in terms of the rates of true and false negatives and positives (as this test had not been validated as a health test), and of the information delivered to consumers.

**Table 1:** Series dates, amounts and names of the main investors in 23andMe [1]

Series	Month/year	Level of investment (millions of dollars)	Invertors
Series A	May 2007	9	Google, Genentech, Mohr Davidow Ventures, New Enterprise Associates
Series B	June 2009	12.5	Google, Sergey Brin
Series C (1)	November. 2010	22	Johnson & Johnson Development Corporation, New Enterprise Associates, Google Ventures
Series C (2)	January. 2011	9	Johnson & Johnson Development Corporation
Series D	December. 2012	50	Google Ventures, Yuri Milner, MPM Capital, New Enterprise Associates, Sergey Brin, Anne Wojcicki

Beyond the legal issues, we focus here on the original nature of the service provided to consumers and its probable consequences in the near future. Indeed, given the exponential nature of the ‘geneticization’ of medicine and medical research [3] in the US and Europe over the last decade, requests for DNA and biological samples from research laboratories have greatly increased in number. The interface between patients and research remains complex and potentially conflictual, due to ethical issues relating to the ownership of the body and of any data pertaining to it, which are still under debate. In this context, a simple but innovative way of proceeding may have emerged. Given how difficult it is to obtain biological samples from a large cohort with the consent and full history of the patients in a short space of time by the standard route, the idea of creating an interface between individuals and researchers has emerged. Indeed, information about human genomes, in addition to being a personal source of useful information for treating the sick or for healthy people that might become sick, could be exploited and used as a source of profit for companies. This vision raises profound ethical issues about the way in which subjects are included in research and about how information about them is gained and used.

An analysis of the 23andMe website and the scientific literature highlights how this and other American biotech companies have specialized in medical genetics so as to become essential intermediaries between researchers and their research subjects, through the generation of DNA banks and biobanks containing hundreds of thousands of different samples provided for DTC genetic testing.

## Discussion

### 23andMe: the two-sided market model

**Fig. 2:**
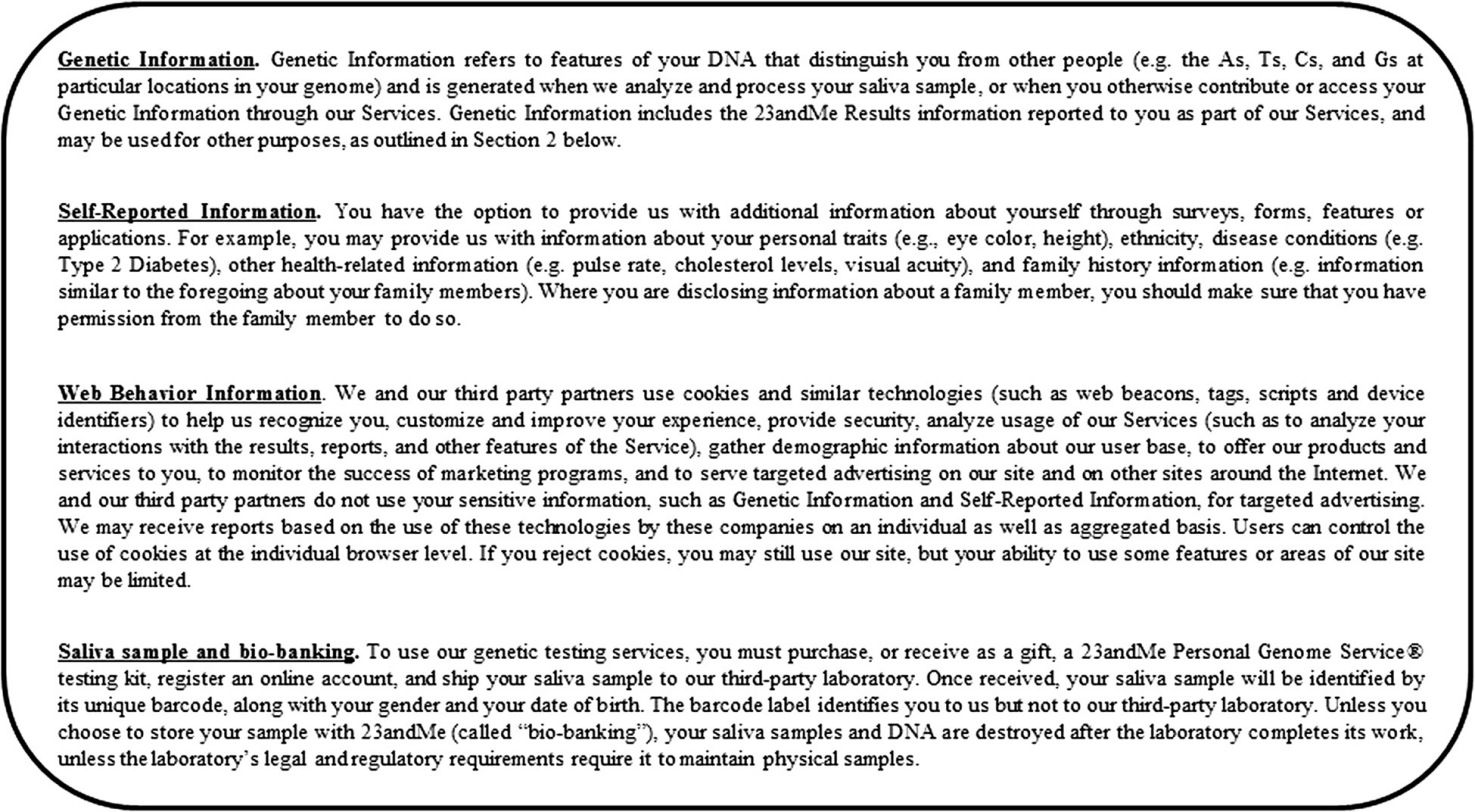
Definitions according to the “Full Privacy Statement” section of the 23andMe website

According to the 23andMe website, particularly the “Terms of Service” section, two types of service seem to be on offer: DTC genetic testing service and participation in “23andMe Research”. The DTC genetic testing service is the service most highlighted and best understood on the website and on social networks (mostly Facebook and Twitter). As explained above, DTC genetic testing is based on the use of SNPs to identify genetic markers associated with specific diseases and conditions and a few specific causal variants. This test is a type of whole-genome analysis (WGA) carried out with a microarray, based on a technique different from whole-genome sequencing (WGS) or whole-exome sequencing (WES), in which all the nucleotide sequences of the genome (WGS) or exome (WES, the exome being the sum of all the exons present) are determined. Interestingly, 85 % of all known disease-causing mutations are in coding regions (detectable by WES) [4]. The genetic information obtained by testing is made available to the consumers via their own protected personal 23andMe web accounts. The company states that its consumers are protected by federal law, under the Genetic Entitled Information Non-Discrimination Act (GINA). This law, in its current state, protects Americans against discrimination on the basis of genetic information. However, according to the “Consent and Legal Agreement” (commercial contract) and the “Research Consent Document” (informed consent), consumers can agree to their genetic information, web behavior information and self-reported information (Fig. 2) being used in the 23andMe Research program, the second service on offer.

23andMe Research could be considered as a voluntary and optional service of the DTC genetic testing service. According to the “Terms of Service”, this service is different from their Research and Development activity (R&D), and is designed to improve services and to offer new products or services to consumers. According to the “Full Privacy Statement” section, “23andMe Research refers to scientific research conducted by 23andMe or third parties in collaboration with 23andMe”. The “third parties” are “public, private, and government partnerships to develop research and advance genetic understanding”. Consumers must sign the informed consent freely available from the company’s website if they wish to make use of this service. This option is associated with the notion of a “full service”, because the company announces that it involves the supply of additional beneficial information not available to consumers making use of the DTC genetic testing service only and not providing informed consent. The third parties are the other side to the 23andMe Research offer. Indeed, in exchange for information about their genes, the consumers supply not only money, but also web behavior information and self-reported information. All of this information is shared with or sold to third parties for the purposes of scientific research and commercial applications, patents or operating licenses. Ethically, 23andMe seems to be at the center of a flow of information between people and research.

**Fig. 3:**
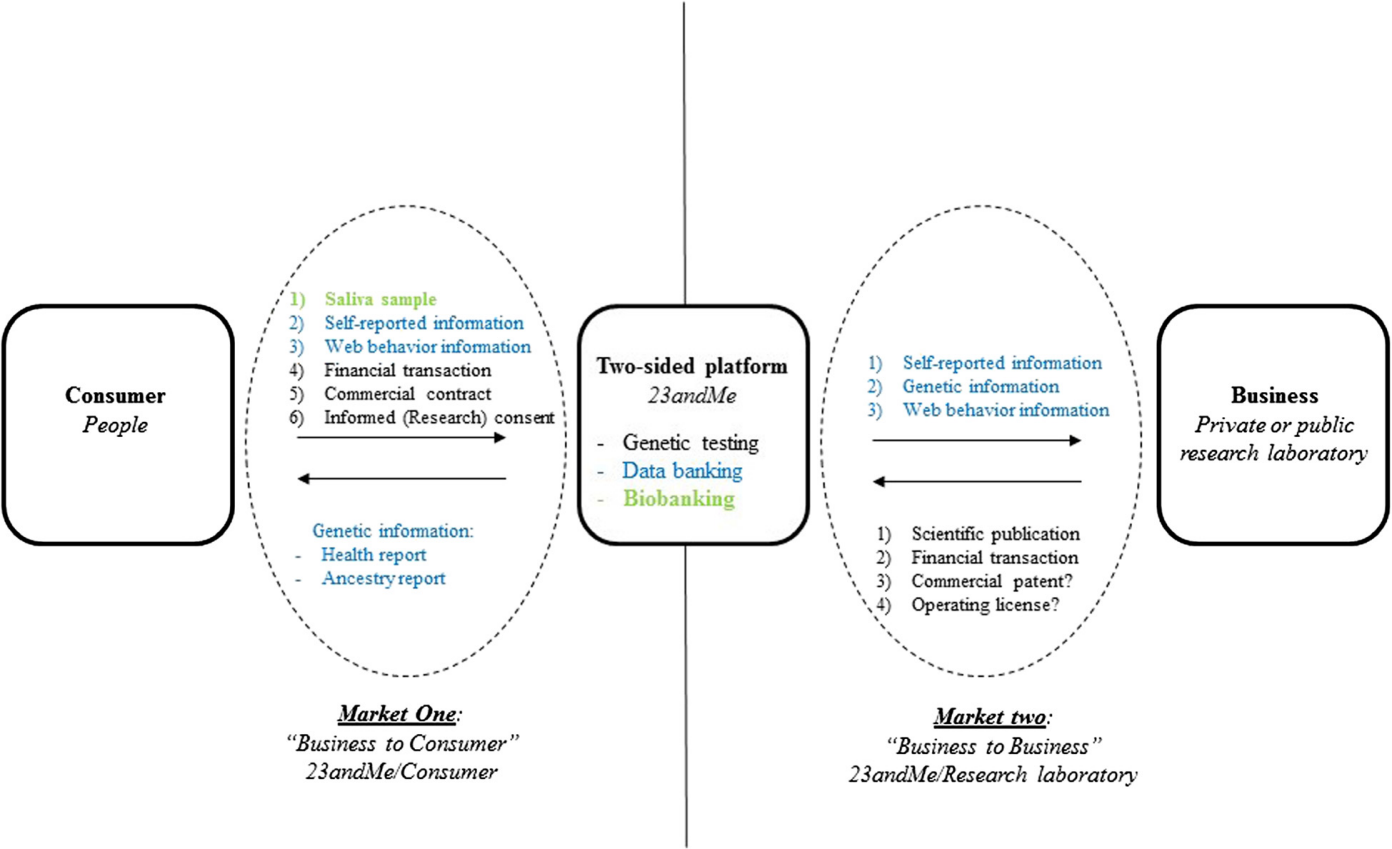
23andMe two-sided market model. Information flows relating to the consumer and his/her body and the database are shown in *blue*. Biological (saliva) sample flows and to and from the biobank are shown in *green*

**Fig. 4:**
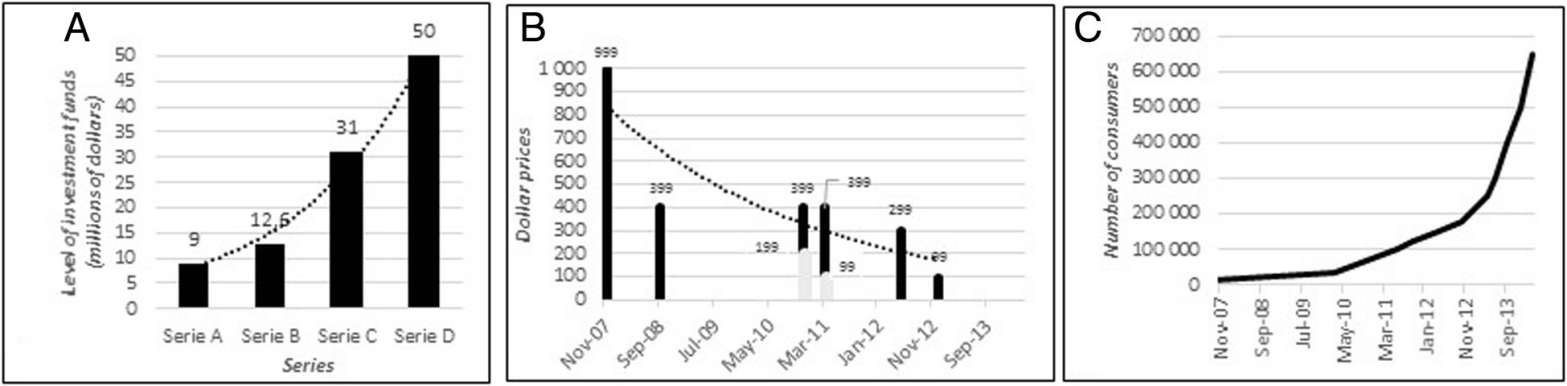
**a** Increasing investment in 23andMe over time. Decrease in kit price over time. November 2010: $399 or $199 + $5 less (at least one year). March 2011: $399 or $99 + $9 per month (one year). **b** The almost exponential increase in the number of users coincides with the falling price of the kit to $99 in November 2012 [1]. **c** Increase in the number of users over time

Can 23andMe be considered to constitute a two-sided market strategy? By definition, a two-sided market model is a market in which two different user groups interact via an intermediary economic platform, known as a “two-sided platform” [5–7]. This set-up makes possible exchanges that would not otherwise have occurred, creating value for both sides. Both sides (in this case, the people seeking DNA analyses and the structures seeking to obtain information about them) can be considered to be consumers. Two-sided markets exist in many different types of industry, occupying the same economic space as traditional offers of products or services, such as those provided by Facebook, Sony or Google (Alphabet), for example. Such a market may exist in the case of 23andMe, and may, indeed, always have been planned as a business strategy. This company may effectively constitute a two-sided platform, with two kinds of consumers: people who want information about their own genes (for multiple reasons), and researchers and others who want access to genetic, web behavior and self-reported information for a large number of people (Fig. 3). The use of this strategy enables 23andMe to obtain biological samples, DNA samples and accurate self-reported information for DNA and biobanking (Fig. 2), with probable strong positive-feedback effects on their business model in the long term. The term “data-banking” does not appear in the informed consent document or in any other document on the website, including the “Privacy Highlights” and the “Terms of Service” (www.23andme.com, US, UK, EU and Canada). The term “bio-banking” is used once, but only to describe the storage of saliva samples (Fig. 2). Three separate events have favored this approach: growing financial investment, falling prices of genetic testing and an exponential increase in the number of consumers over a very short period of time (Fig. 4) [1].

### DNA banking issues

In the past, biological samples were stored in a single laboratory [8], but large collections of DNA samples are becoming increasingly common in human genetics. With the publication of the first draft sequence of the human genome in *Nature* [9] and *Science* [10], and its completion by the Human Genome Project, the strategic importance of DNA banking and data collection has increased. Over the last 10 years or so, medicine and medical research have become increasingly “geneticized”, with the general population becoming increasingly interested in genetic aspects. DNA can be obtained from a number of potential sources, including the blood, cell and tissue banks of hospital and academic research centers, and it has been estimated that there are already several hundred million biological samples stored in such repositories [11]. 23andMe do not state the exact number of samples to which they have access on their website or in other documentation, but it can probably be safely assumed that they have a collection of hundreds of thousands of biological and DNA samples. Indeed, their collection may be one of the largest available and it is driven not only by scientific or medical aims, but also by business imperatives [12]. 23andMe have established a high-value biobank and database for use by private and public research laboratories (Fig. 3).

DNA banking is becoming a business and there is a rush to acquire DNA sequencing data, which may prove to be the organic and molecular equivalent of a gold mine. With personalized medicine, biofuels and genetically modified organisms (GMOs), the potential gene market in 2030 is estimated to be worth as much as $100 billion [13]. In 2001, the technology used to sequence the human genome was still based on capillary electrophoresis of individual fluorescently labeled Sanger reaction products; it produced 115 thousand base pairs per day [14] for a total cost of almost $3 billion [15]. For 30 years, until the advent of next-generation sequencing (NGS) methods, “Sanger sequencing” was the only method used for DNA sequencing [16]. Devices for NGS first appeared in 2007 and owe their success to a synchronous sequence analysis, resulting in faster, more sensitive analyses at a lower overall cost [17]. Indeed, whereas Sanger’s direct (or first-generation) sequencing method requires the generation of DNA strands of different lengths labeled with a fluorophore for analysis, NGS methods reconstruct previously prepared DNA strands by direct determination of the nucleic acids incorporated [18]. These methods, based on a sequencing-by-synthesis approach, have increased sequence output per run and read length, and have decreased costs and improved the accuracy of base-calling [14]. With the release of the HiSeq X Ten, the genetic sequencing company Illumina is currently attempting to solidify its domination of the market, with the possibility of sequencing an entire genome for $1,000 [15]. However, this offer is not yet available to everyone, as the HiSeq X Ten system is available only as a combination of at least 10 HiSeq X machines, with each machine costing around $1 million (Illumina source). This system could sequence the genomes of 18,000 humans per year. However, even if DNA sequencing costs are declining, questions still remain about the generation, storage, analysis and interpretation standards for genetic data. This issue is of particular relevance for DTC genetic testing, even if WGA by genome-wide SNP chip approaches are subsequently replaced by WGS.

For DNA sequencing, a DNA source is required, and interpretation of the genetic data generated requires information about the source, such as clinical data or private data for the patient. Obtaining more information about the source improves the quality of interpretation for genetic data and, thus, their scientific and medical value. However, these new approaches require even faster technology, as previously reported. In 2012, Oxford Nanopore Technology (ONT) introduced a new sequencing system called Minion, based on nanopores (pores with a diameter between 1 and 100 nm). This technology has two advantages: sample preparation is very simple and does not require expensive reagents and longer reads can be obtained (>1,000 bp). However, the error rate (4 %) is currently too high for most of the applications envisaged, including medical diagnostics. Nevertheless, this technology may, in the near future, provide real new opportunities for DTC testing services. Indeed, in medicine and industry, the key issue is the relationship between specific genetic sequences, not necessarily restricted to SNPs or potential causal mutations, and particular diseases, with a view to guiding treatment and developing new drugs. This relationship is the key to the financial value of DNA data and may be the premise underlying the development of two-sided platforms, such as that of 23andMe, for obtaining large numbers of samples and considerable amounts of information for research and industry through a DTC genetic testing service. These approaches are based on DNA banking, but DNA sample collections may differ considerably in several critical ways: storage, confidentiality, requests, security and quality [19]. These aspects and the differences in them between collections raise ethical issues.

### Ethical aspects of the model

**Fig. 5:**
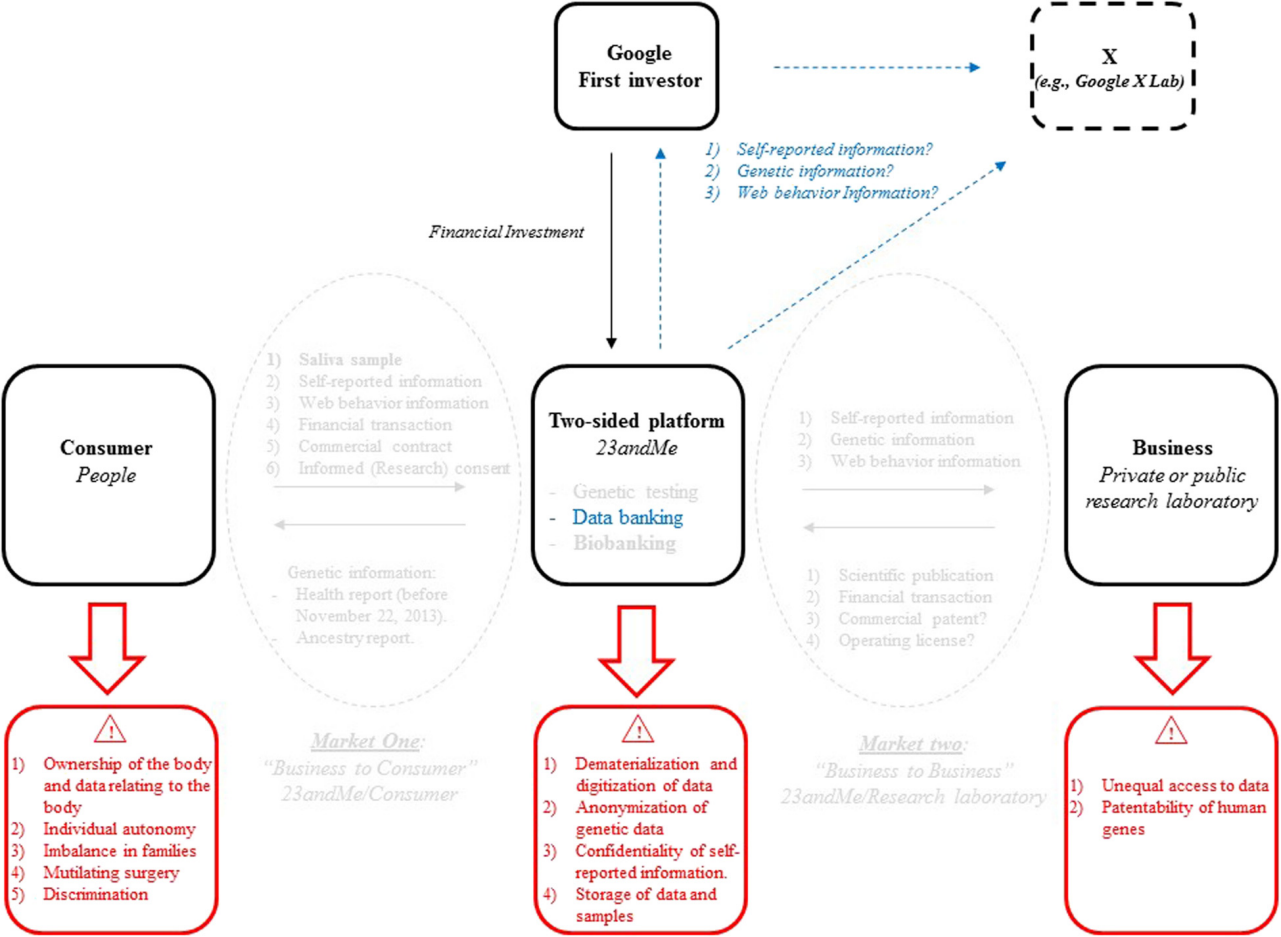
Ethical aspects of the 23andMe model and connections with Google. Information flows relating to the consumer and his/her body and database are shown in *blue*. Ethical issues are shown in *red*

The two-sided market model of 23andMe can be considered a case study. This model raises important ethical questions about genetic testing, DNA banking and research relating to autonomy, ownership of the body, data obtained from the body, and informed consent (Fig. 5). The people sending samples are not considered to be patients. They are instead considered to be consumers, and the majority are healthy. They are given the opportunity to give informed consent for participation in scientific research, but they primarily sign a commercial contract and pay for the purchase of a service, offering genetic testing. When obtained, consent is not given during an individual medical consultation with a physician or a genetic counsellor at a hospital or in a doctor’s surgery. The clients give their consent alone, via their computer, at home. This set-up raises a major ethical issue: that of the autonomy of the individual, in particular, and the right to access to his or her own genetic information [20]. In France, oral information explaining the goal of testing is considered necessary. According to French civil law, the individual does not own his or her body and cannot ask directly for genetic health tests to be directly. Furthermore, it remains a matter of debate whether individuals really have the right to access their own genetic information, their own DNA [21, 22]. Instead, a physician must prescribe the test, with the approval of the “*Agence de Biomédecine”* (Biomedicine Agency). Moreover, the test cannot be performed by a private company, but only by a public genetics laboratory also approved by the “*Agence de Biomédecine”*. A medical diagnosis must be based on the sequencing of specific genes to be considered accurate. The need to protect privacy is illustrated by the family imbalances, preventive and curative surgery following diagnosis and discrimination by insurance companies that may result from poor regulation [23].

Several studies have focused on genetic tests and raised the issue of the apparent ethical conflict between two concepts of autonomy: some specialists from different countries prefer to maximize autonomy, whereas others support the notion that autonomy is only effective if accompanied by protective measures [24, 25]. Some authors have suggested that one of the prerequisites for autonomous choice is that the person is able to understand the information provided and to provide a rational argument for his or her choice [26–28]. The degree of autonomy of the customers of companies such as 23andMe is highly variable, depending on genetic competence [29]. Indeed, we can assume that the individual’s knowledge of genetics, family history and the penetrance or pathogenicity of the disease will determine his or her ability to evaluate the benefits and risks of genetic testing and the consequences for members of his or her own family, to compare tests prescribed by a physician and non-prescribed tests and to compare the different DTC testing companies in terms of the quality of the services on offer.

Two major problems relating to autonomy are immediately apparent here: the problem of scientific literacy [30] and the lack of training of physicians in genetics [31, 32]. Indeed, it has been shown that more than half the individuals buying genetic tests online subsequently consult a physician to discuss the result [33]. It might therefore be a good idea to create a “direct-to-physician genetic reporting service”, or at least an optional service of this kind, effective before and after the purchase of the kit. Such a service would ensure that physicians were better trained in the interpretation and explanation of genetic tests and would ensure better counseling and follow-up for users, while providing users with greater autonomy. It would also make it possible for the test offered by 23andMe to be considered a real medical diagnosis test rather than as simply providing information. This vision of autonomy differs from those prevailing in the UK and the US, in which all individuals wishing to have access to their genetic and medical data are free to do so, provided an agreement has been reached with the family in cases of clinical analysis, regardless of their level of genetic knowledge and the conditions of the service on offer [29, 34].

The press has reported incidents in which families requesting genetic tests via the Internet have learned, from the results obtained, that the presumed father was not the real biological father or in which the biological father has found that he has children that he didn’t know existed [35, 36]. 23andMe warns its clients in advance of the possibility of such discoveries being made through its tests (see the website and blog of 23andMe) and seems to have resolved this problem. It nevertheless remains legitimate for civil society to pose the question as to whether private companies should be able to reveal such information through so-called “phylogeny” or “health report” genetic testing rather than through (official) paternity tests, particularly if they do not ensure, other than virtually, that this choice was consented to by all of the people concerned by the results and not just by the principal person concerned [34]. For preventive surgery, the best known case is undoubtedly that of an American actress who underwent a prophylactic double mastectomy following positive results in a genetic test for the breast cancer 1 (BRCA1) mutation [37]. In the wake of her decision, an increase was observed in the numbers of BRCA1 and 2 tests and of prophylactic double mastectomies carried out [38]. Over and above the principle of not doing harm, which is called into question by major, potentially traumatic surgery, this approach also raises questions about the principle of autonomy, because there may be a risk of abuse in the long term. Indeed, if such practices were to become systematic, insurers might have the right to oblige their clients to undergo testing if they have a family history of disease, particularly for genetic predispositions to cancer, due to the costly and debilitating nature of targeted treatments if the disease is diagnosed late.

Ethical issues also arise within the testing company. These issues include the dematerialization and digitization of data, the anonymization of genetic data, the confidentiality of self-reported information and the storage of data and samples (Fig. 5) [39, 40]. Indeed, when informed consent forms and the commercial contract are signed digitally, the consumers provide the company with their names, together with a set of personal information about themselves and their families relating to health and ethnicity. The company states on its website that names, addresses, e-mail addresses and bank data will not be disclosed or used. Nevertheless, such information (Fig. 2) significantly increases the medical, scientific and financial value of the data. It is therefore unsurprising that the contract stipulates that data, DNA and biological samples will be kept and may be re-used in other research if the consumer consents. With the multiplication of this transaction by hundreds of thousands of individuals, the company is well aware that its consumers have not only paid a few hundred dollars each, on average, for genetic testing services, but have actually sold their samples and information for inclusion in a major biobank and database for use by scientists and doctors. But what are the consequences for the consumer? Can separate pieces of information about an individual be brought together? Particularly as concerns the consumer’s name and important items of personal information? These issues have not been sufficiently explored by the company, which seems to safeguard its own interests more strongly than those of its consumers, although 23andMe received institutional review board approval for its research protocol and a revised consent document in 2010 (23andMe blog). Indeed, Genentech has paid $60 million (in total) for the WGS data of 3,000 23andMe consumers with Parkinson’s disease, with the aim of generating new therapeutic target leads [41]. However, despite large amounts of clear, illustrated information about the phenotypes and/or diseases revealed by their analyses, the robustness and accuracy of the chip used for testing are not perfect at individual level and not all of the mutations detected have been validated by Sanger sequencing (thereby potentially mixing false and true positives and negatives). A client may therefore unknowingly carry a deleterious mutation that may be reported in the information delivered by the company or may knowingly carry such a mutation that is not identified in the results delivered by the company. The risk of false-positive or false-negative results for these tests is the principal concern of the FDA and its approval process [42]. However, in the context of a study of several thousand people (carried out by GWA), missing a single SNP in an individual is not a problem because there are thousands of others. At an individual level, missing a SNP may have much greater consequences. The previous economic model was based on low prices to attract more consumers, providing more data and biological samples to be valorized and sold. The four rounds of investment in this company (Fig. 4) may have been designed to address the problem of the probable lack of benefit from this side of the market until the second market had been established (Fig. 3) [43]. This second market has now been established through the collaboration between 23andMe and Genentech.

According to a French *Agence de Biomédecine* report on genetic tests published in 2014 [44], there is currently no consensus definition of a genetic test and very few countries have adopted specific legislation relating to genetic testing, the principal countries to have done so being Austria, Switzerland, Germany and Portugal. In the United States, genetic tests for medical purposes are accessible without a medical prescription and are billed by the laboratories concerned. However, 24 American states have prohibited divulgation of the results of genetic tests in the absence of a physician. Nevertheless, companies selling genetic tests via the Internet, such as 23andMe, at least before they were prevented from doing so by the FDA, report the results of their tests directly to their clients. According to an *Institut National de la Santé et de la Recherche Médicale* (INSERM) report on genetic tests dating from 2008 [45], this is permitted because genetic information is not considered to be particularly sensitive in the US. Instead, it is seen as ordinary personal information, unless supplied by a genetic test governed by the FDA. Nevertheless, increasing numbers of “genetic privacy” laws have been passed in the US in recent years, by contrast to Europe, which now seems to be moving in the opposite direction [44]. Indeed, the European Union (EU) is increasingly moving towards the broader and freer circulation of data for research purposes [44]. According to the French *Agence de Biomédicine* report published in 2014 [44], the UK has recently gone further, because the Human Genetics Commission responsible for providing the British government with expert advice now considers that it would not be desirable to ban tests bought over the Internet, simply because it would be impossible to police such a ban given the freedom of access to the Internet available today. However, it did recommend the establishment of a certain number of guidelines concerning test quality, the information transmitted and the qualifications of those carrying out the test. The proof of this shift in position is that 23andMe entered the British market in December 2014, about a year after it was banned by the FDA in the US [41, 46]. Ireland, Denmark, Finland, the Netherlands and Sweden have all accepted the sale of the 23andMe test in their territories (23andMe Europe). It might be possible for France to follow the same course of action. Most countries are currently facing a change in the definition of health data much more complex than any previously observed, which has been neglected for far too long. It is now possible to generate health data with non-certified medical technologies as simple as a smartphone or any kind of connected object [47]. Is it really the fault of 23andMe for having understood this issue or that of the health authorities for not having thought sufficiently deeply about it?

The data and samples that 23andMe “lend” to different research teams also raise two other ethical problems: the inequality of access to data and samples between research teams due to differences in financial resources or nationality, as the laws of some countries are not compatible with the patentability of human genes (Fig. 5.). One direct consequence is a significant bias in the race for publication and international tenders [39]. The issue of the patenting of human genes is far from resolved, particularly in the US and in European countries, such as France. In the emerging world of targeted molecular therapies and genetic tests for diagnosis and prognosis, these questions will need to be addressed [39, 48]. There are already inequalities between research teams in terms of the production and use of scientific knowledge, through access to high-quality scientific publications or various new technologies, probably due to significant qualitative and quantitative differences in resources between countries and between research teams in the same country. Should access to and use of knowledge be based on the financial clout of a team or its scientific intuition, particularly if society hopes for new ideas to emerge from science [49]?

Another link that would merit closer scrutiny is that connecting 23andMe to Google. Google was one of the principal investors in all four rounds of financial investment in this company. Google may be interested in the web behavior information for its search engine activity, and in the self-reported information and genetic information, which may be of use to subsidiary companies, (e.g., X, originally Google X lab) (Fig. 5). This idea raises other ethical issues due to the transhumanist vision of Google, with its growing monopoly on the emergence of new technologies, ultimately resulting in a lack of competition and, in some instances, a possible threat to democracy. Indeed, after five years of investigation, the European Commission accused Google of abusing its dominance of the market in April 2015 [50]. This example illustrates the difficulties inherent to companies attempting both to provide services and to relay information. 23andMe should therefore carefully consider the risks they face, because, in this instance, the products are biological materials and health data.

## Conclusion

23andMe had the brilliant and original idea of responding to the desires of the population at a large scale. In accordance with legislation, they offer consumers genetic data obtained with current technology and they aim to create a valuable biobank with data and DNA from 1,000,000 individuals. This new two-sided data-banking market is developing more rapidly than consumer protection laws. A profound ethical reflection on the practices of this new market model is therefore required, taking into account the history of 23andMe and other companies. By communicating more openly with clients and with the press about its data-banking activities, 23andMe might help the wider community to perceive the benefits of such a large-scale donation of data (genetic, medical and personal) to companies through the purchase of a kit. Greater communication would also increase transparency concerning the second market, which is essential for the development of a lucrative data-selling enterprise, as it would suspend the distrust and interrogations (some based on pure fantasy) of consumers, the scientific community and the press. As expected, following the provision of more information and tests to the FDA, 23andMe was granted FDA authorization on February 19, 2015, first to provide reports about Bloom syndrome carrier status [41], and, more recently, to provide consumers with “carrier status” information for 35 genes known (with high levels of confidence) to cause disease [51]. Further authorizations are likely to follow, because ethical reflections on testing practices are now underway.

In conclusion, full information and an open mind are essential to prevent misunderstandings. This synergy between 23andMe and its research and commercial partners may extend well beyond what the consumers initially sought, with predictions for the future in which DNA molecules are used like crystal balls. It could result in new class actions, like those already experienced by the company. We may now have no choice but to deal with a new form of two-sided market model that is likely to inspire other companies in the near future. For these reasons, it is essential to anticipate developments through the ethics reflections proposed by European and Canadian bioethicists [19, 22], to help these companies to grow and to allow scientific research to progress, while maintaining the best possible protection of individuals.

We think that 23andMe should provide more information about its data banking activities to its clients and the press so that society can be made more aware of the benefits of such a donation of data (genetic, medical and personal) through the sale of the kit via the Internet. We recommend greater transparency concerning the second market the sale of the data obtained. If such an approach had been employed from the outset, much suspicion and many questions (some entirely based on fantasy) from consumers, the scientific community and the press might have been avoided, together with the class action against the company. However, companies such as 23andMe are raising key questions that we need to address, concerning autonomy, ownership of the body, data relating to the body and informed consent in the new digital age. It has now become necessary to define new rules validated by all for the use of different balances between consumers and patients, the services offered and private and public healthcare, freedom and justice in a democratic, liberal and globalized background, if we wish to construct a new world in which the patient is fully respected by innovative genius, enterprise and international law.

### Ethics approval and consent to participate

Not applicable.

### Consent for publication

Not applicable.

### Availability of data and materials

Not applicable.

## Abbreviations

BRCA1: Breast cancer 1
BRCA2: Breast cancer 2
DNA: Deoxyribonucleic acid
DTC: Direct-to-consumer
EU: European Union
FDA: Food and Drug Administration
GINA: Genetic Entitled Information Non-Discrimination Act
GMO: Genetically modified organism
GWA: Genome-wide association
INSERM: *Institut National de la Santé Et de la Recherche Médicale* (French National Institute of Health and Medical Research)
NGS: Next-generation sequencing
ONT: Oxford Nanopore Technology
R&D: Research and Development
SNP: Single-nucleotide polymorphism
UK: United Kingdom
US: United States
WES: Whole-exome sequencing
WGA: Whole-genome analysis
WGS: Whole-genome sequencing
